# Supplementary figures and images for: Lower DNA methylation levels in CpG island shores of CR1, CLU, and PICALM in the blood of Japanese Alzheimer’s disease patients
Source: PLoS One. 2020 Sep 29;15(9):e0239196. doi: 10.1371/journal.pone.0239196 (PMC7523949; doi:10.1371/journal.pone.0239196)

CR1 (-2,000~146,501)

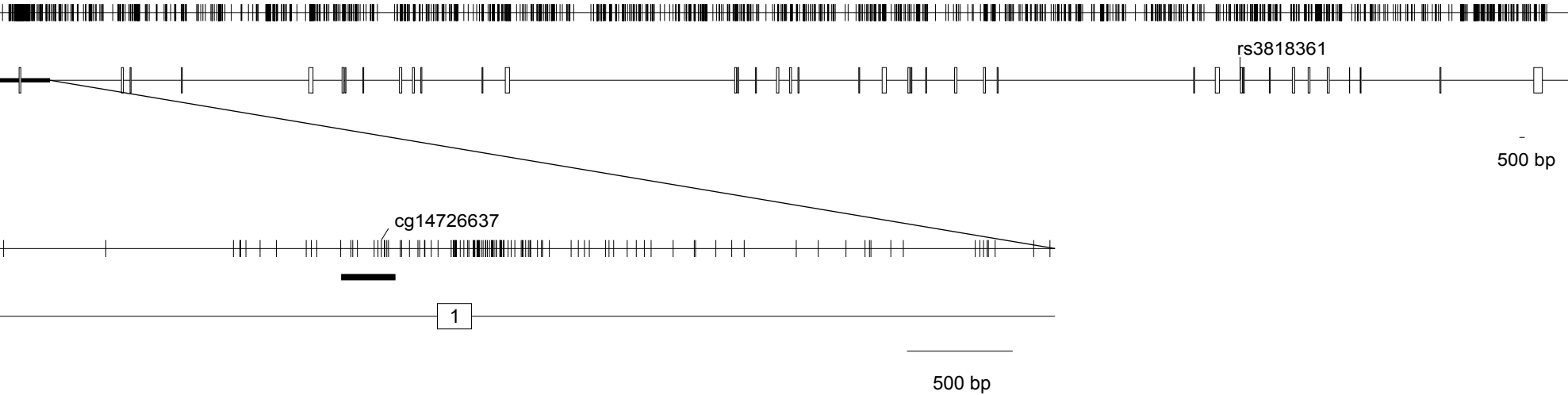

Supplement: S1 Fig — The schematic shows the distribution of CpG dinucleotides along CR1. The position of the transcription start site is defined as +1; hence, “-2,000” in the parenthesis indicates that the diagram includes 2 kb upstream of CR1, whereas “146,501” in the parenthesis is the position 2 kb downstream from the end of the last exon. Vertical lines indicate the positions of CpG dinucleotides. Open rectangles depict exons and a region spanning the first exon is enlarged. The fundamentals of these diagrams were automatically drawn by the web-based tool CyGnusPlotter. It collects the genomic structure of the most representative isoform of a requested gene from the Ensembl database with upstream and downstream regions of the designated lengths. Underlined is the region for which the methylation level was analyzed by bisulfite sequencing. It included a CpG to which Illumina designed a probe for the 450k array and gave the ID “cg14726637”. The position of the AD-associated SNP, rs3818361, is also shown. The thick horizontal line represents the position of amplicons for bisulfite sequencing. (PDF) [file pone.0239196.s001.pdf]

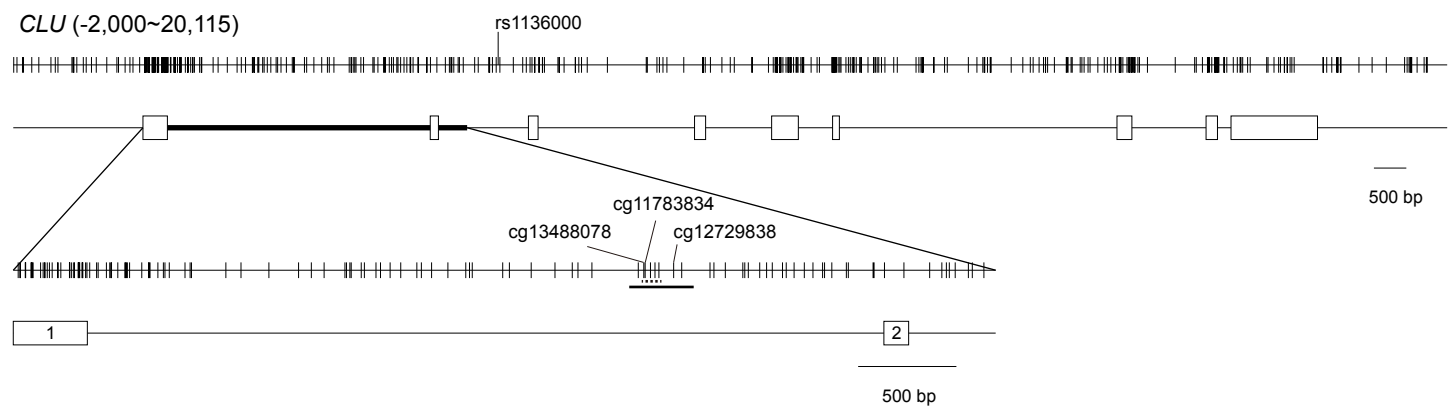

Supplement: S2 Fig — The figure is drawn similar to that in S1 Fig, except for the dashed line, which indicates the region in which methylation levels were quantified by pyrosequencing. (PDF) [file pone.0239196.s002.pdf]

*PICALM* (-2,000~114,198)

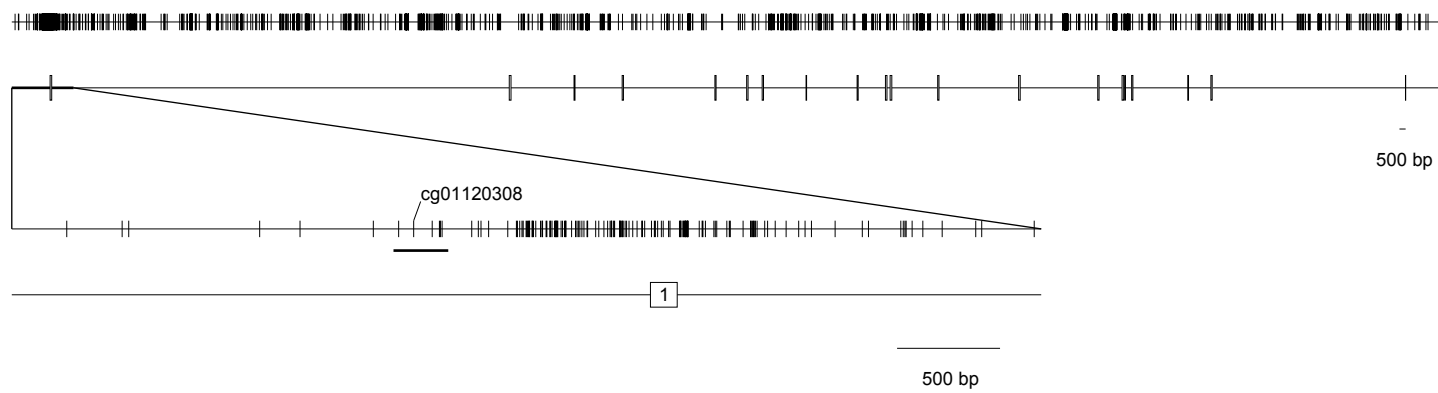

Supplement: S3 Fig — The figure is drawn similar to that in S1 Fig. The AD-associated risk SNP, rs3851179, which is linked to the gene, is not shown in the figure because it is located ~80 kb upstream. (PDF) [file pone.0239196.s003.pdf]

*ABCA7* (-2,000~27,467)

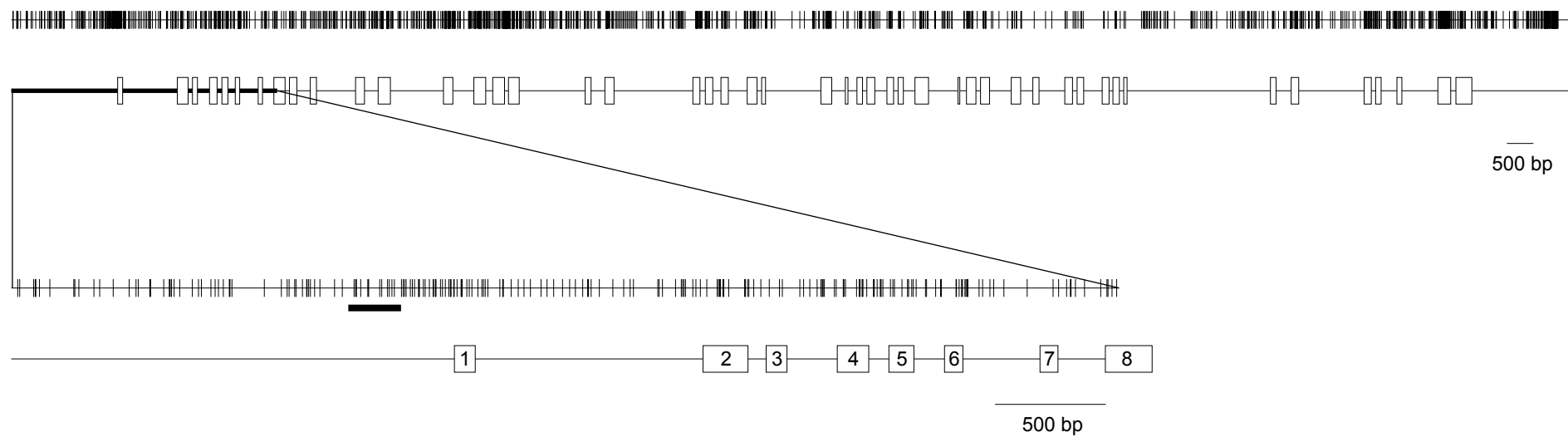

Supplement: S4 Fig — The figure is drawn similar to that in S1 Fig. (PDF) [file pone.0239196.s004.pdf]

*BIN1* (-2,000~61,329)

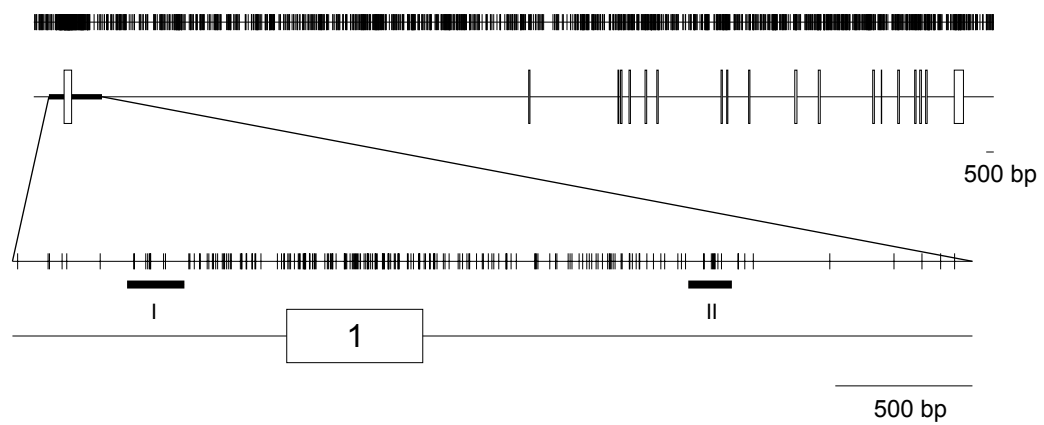

Supplement: S5 Fig — The figure is drawn similar to that in S1 Fig. In this gene, there are two regions (I and II) for which methylation levels were quantified by bisulfite sequencing. (PDF) [file pone.0239196.s005.pdf]

*TREM2* (-2,000~6,680)

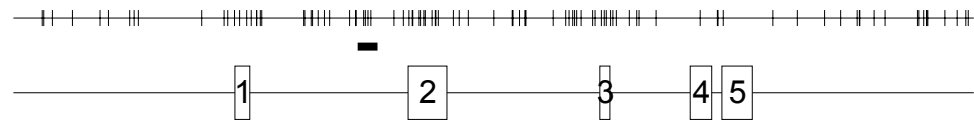

500 bp

Supplement: S6 Fig — The figure is drawn similar to that in S1 Fig except that the underlined region is the region for which methylation levels were analyzed by pyrosequencing. (PDF) [file pone.0239196.s006.pdf]

## Slide 1
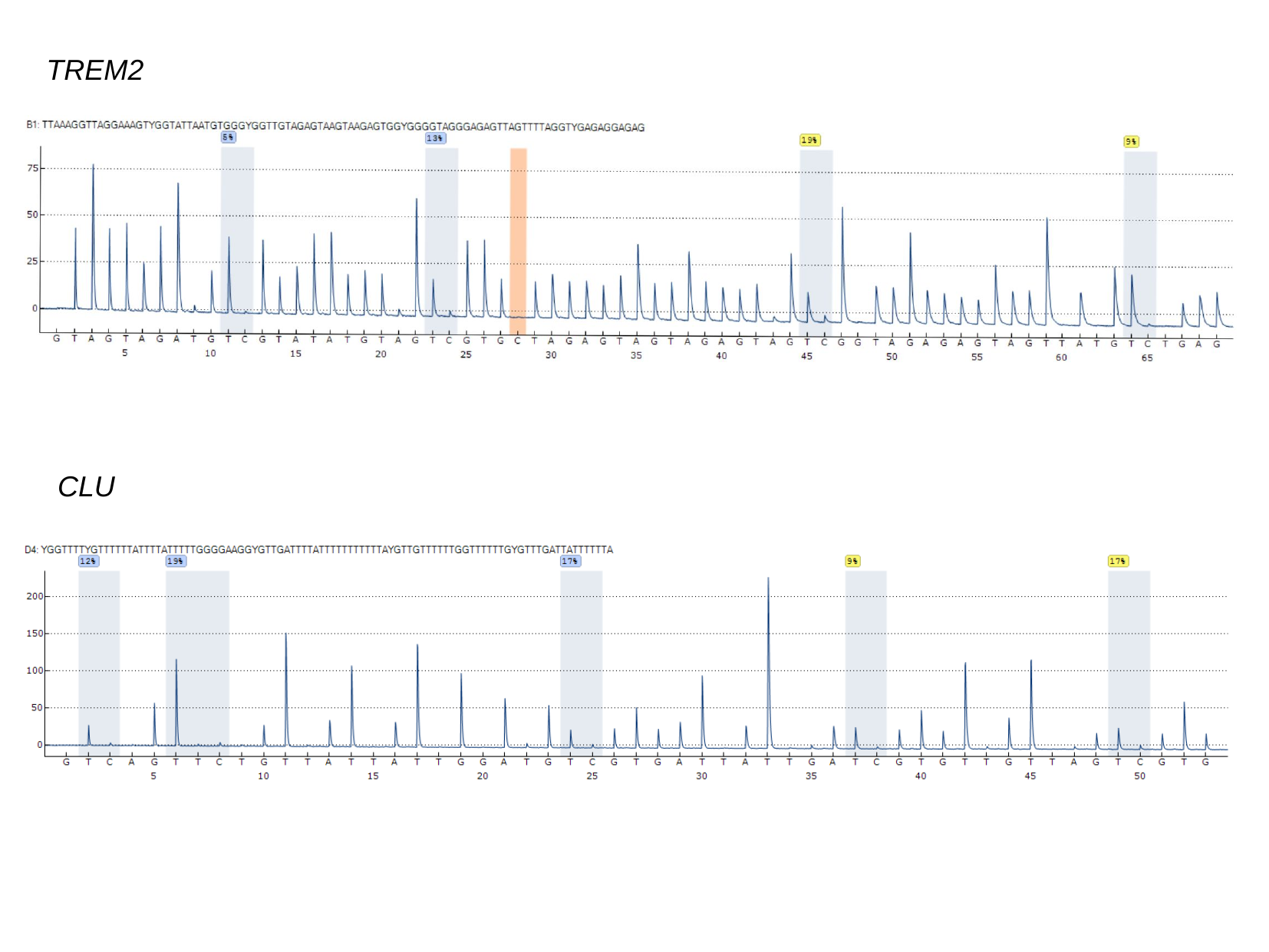

TREM2
CLU

Supplement: S7 Fig — The plots shown are PyrogramsTM, automatically generated by PyroMark Q48 Autoprep Software after pyrosequencing. The short sequences in the PyrogramTM show the bisulfite-converted target sequences in which cytosines to be analyzed for methylation are indicated as Y (= C/T). The positions of Ys correspond to grey rectangles in the PyrogramTM. The numbers in small blue and yellow boxes indicate % methylation with high and intermediate sequencing qualities, respectively. The long rectangle in orange covers a cytosine that is not followed by guanine but by adenine in the template sequence of TREM2, which should therefore be converted to thymine by bisulfite treatment and PCR. Full bisulfite conversion was confirmed by no peaks at the cytosine position. (PPTX) [file pone.0239196.s007.pptx]

A

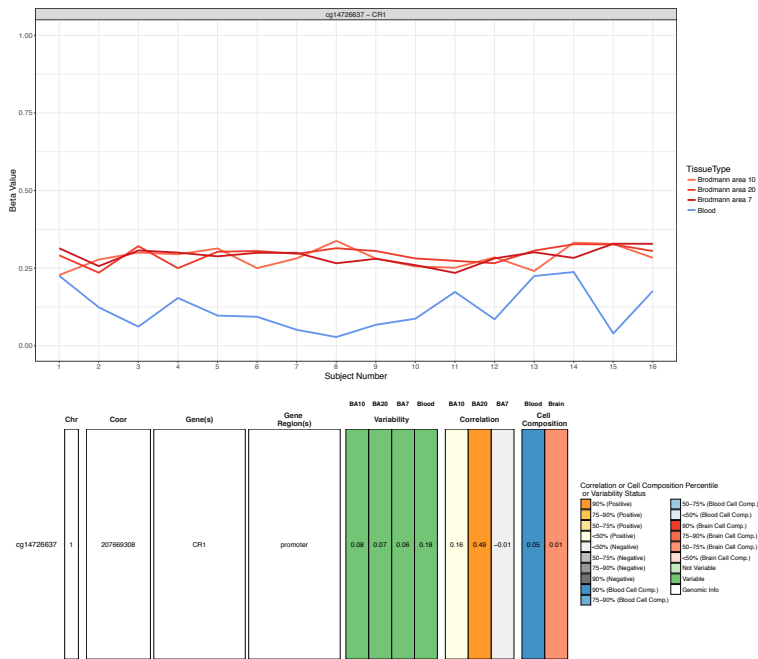

B

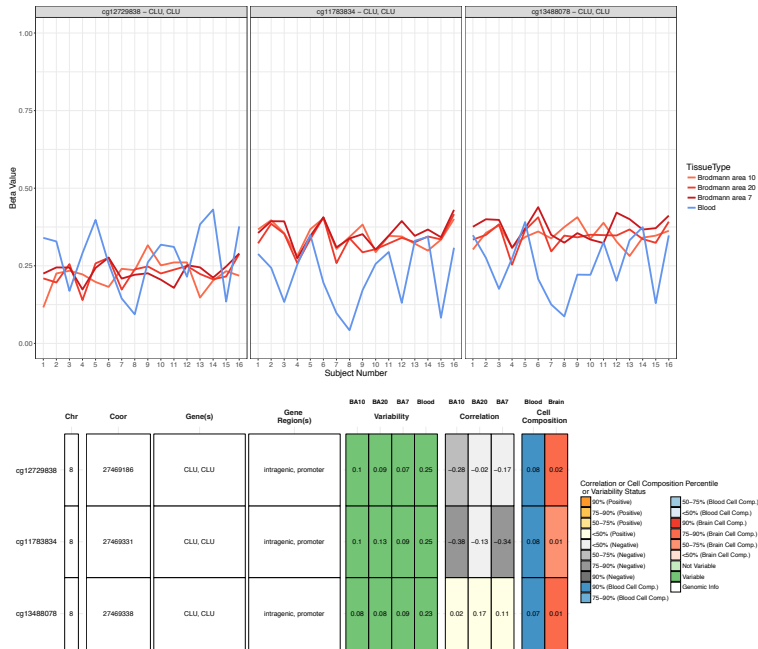

C

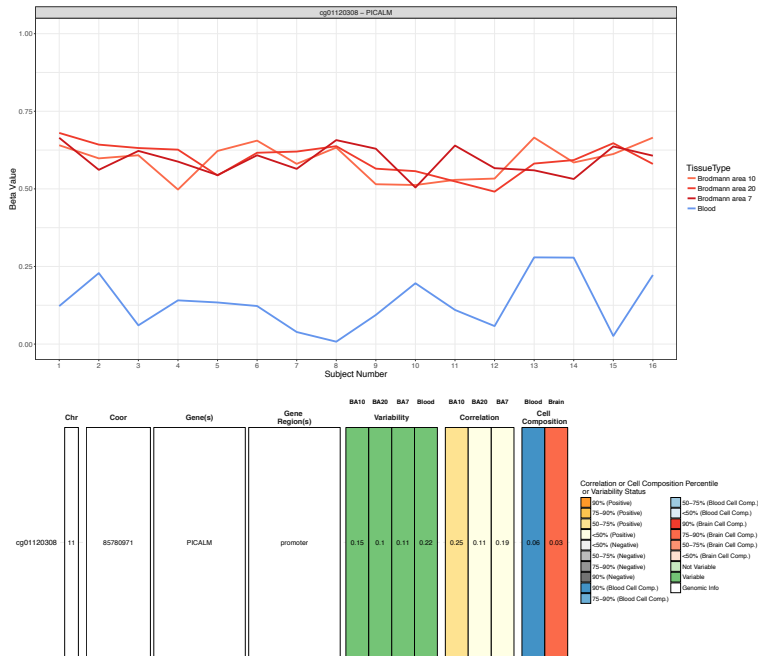

Supplement: S10 Fig — Each plot shows the inter-individual variability of the methylation level at a CpG site across 16 subjects. The five CpGs examined were derived from three AD-associated DMRs identified in the present study: one CpG in CR1 (A), three CpG sites in CLU (B), and one CpG site in PICALM (C). The numbers in “Correlation” columns in the tables indicate Spearman’s rank correlation coefficients (rS or ρ), which were obtained by comparisons of methylation levels between blood and either one of three different cortical regions (Broadmann area 10 (BA10), prefrontal cortex; Broadmann area 7 (BA7), parietal cortex; and Broadmann area 20 (BA20), temporal cortex). (PDF) [file pone.0239196.s010.pdf]

**A**

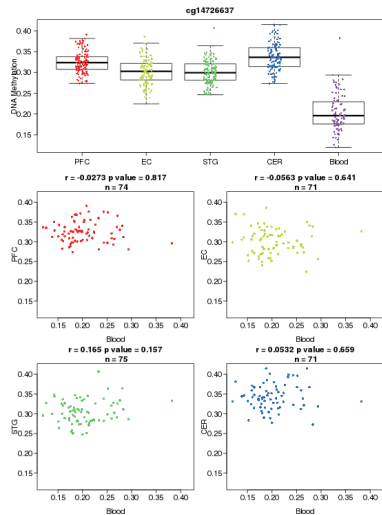

**C**

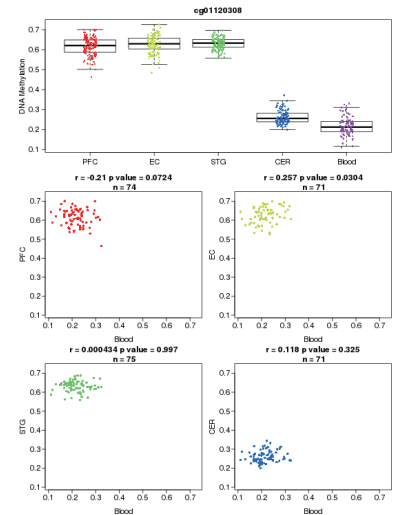

**B**

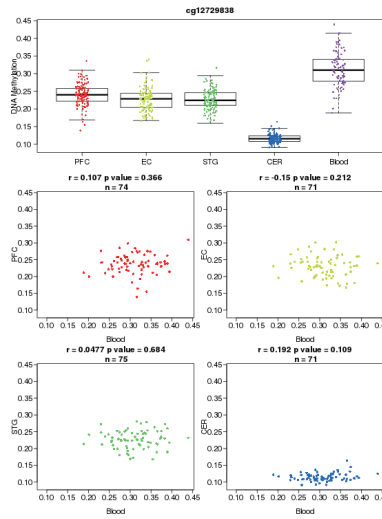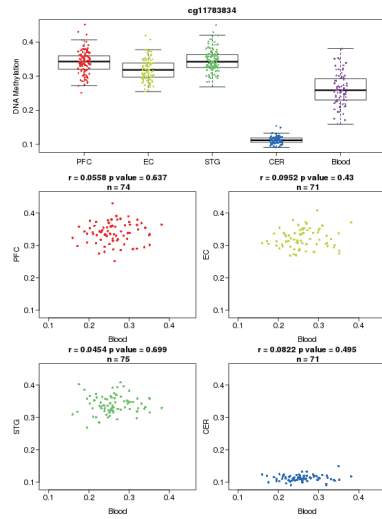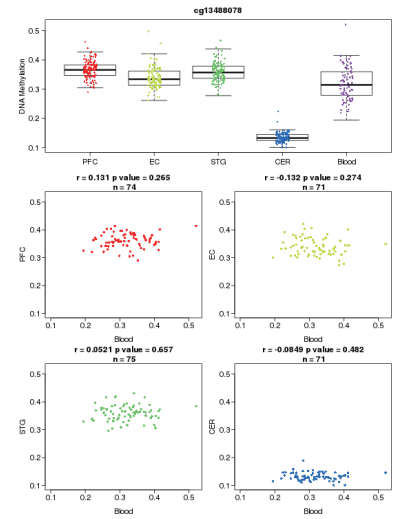

Supplement: S11 Fig — Methylation levels at the five CpGs in S7 Fig, a CpG site in CR1 (A), three CpG sites in CLU (B), and a CpG site in PICALM (C) in blood and four brain regions (PFC, prefrontal cortex; EC, entorhinal cortex; STG, superior temporal gyrus; CER, cerebellum) from the same individual donors were plotted in the rectangles, and the correlation of DNA methylation in blood with the four brain regions are plotted in square boxes. Methylation data were generated by Hannon et al. [40]. (PDF) [file pone.0239196.s011.pdf]
