# Supplementary material for: Lower DNA methylation levels in CpG island shores of CR1, CLU, and PICALM in the blood of Japanese Alzheimer’s disease patients
Source: PLoS One. 2020 Sep 29;15(9):e0239196. doi: 10.1371/journal.pone.0239196 (PMC7523949; doi:10.1371/journal.pone.0239196)
Supplement: S1 Table — (DOCX) [file pone.0239196.s013.docx]

| Gene | Location | Primer name | Primer sequence (from 5’ to 3’) | PCR conditions | No. of CpGs | Product size |
| --- | --- | --- | --- | --- | --- | --- |
| *APOE* | upstream  Fig. 1 (region I) | APOE upst2 CH3 F1 | GTTTTTATTGTGTGGTTTTGTTATT | 95ºC, 10 min.:(94ºC, 30 sec.: 55ºC, 30 sec.:  72ºC, 30 sec.) ×40 cycles: 72ºC, 7 min. | 8 | 256 bp |
|  |  | APOE upst2 CH3 R1 | TCTAAACATCAAATTCCTTTACTCATC |  |  |  |
|  | upstream  Fig. 1 (region II) | APOE 5Alu CH3 F1 | GATTTTTAGTAGGTGTATTATATTGTTTTT | 95ºC, 10 min.:(94ºC, 30 sec.: 53ºC, 45 sec.:  72ºC, 60 sec.) ×40 cycles: 72ºC, 7 min. | 12 | 491 bp |
|  |  | APOE 5Alu CH3 R1 | TTCCTAACCCTATCCTTTCCTATAC |  |  |  |
|  | exon 1 / intron 1  Fig. 1 (region III) | APOE exon1 CH3 F1 | GGGTAGGGGGAGAATAGTTTATTT | 95ºC, 10 min.:(94ºC, 30 sec.: 55ºC, 30 sec.:  72ºC, 30 sec.) ×38 cycles: 72ºC, 7 min. | 9 | 228 bp |
|  |  | APOE exon1 CH3 R1 | CAAAATCCCAACTCTTTCTAAAAAC |  |  |  |
|  | exon 2 / intron 2  Fig. 1 (region IV) | APOE intron2 CH3 F1 | TTGTGTTGTTTTTTGGTTTTGAATA | 95ºC, 10 min.:(94ºC, 30 sec.: 55ºC, 30 sec.:  72ºC, 30 sec.) ×40 cycles: 72ºC, 7 min. | 9 | 260 bp |
|  |  | APOE intron2 CH3 R1 | ATAACATATACCTATAATCCCAACTACTTA |  |  |  |
|  | exon 4  Fig. 1 (region V) | APOE exon4 CH3 F1 | GGTTGTTTAAGGAGTTGTAGG | 95ºC, 10 min.:(94ºC, 30 sec.: 55ºC, 30 sec.:  72ºC, 30 sec.) ×40 cycles: 72ºC, 7 min. | 38 (ε4: 39) | 322 bp |
|  |  | APOE exon4 CH3 R1 | ACCAAAAAACCCACAATAAC |  |  |  |
| *CR1* | upstream  S. Fig. 1 | CR1 up1 CH3 F1 | TTTTAATTTAGGGTAAGGAAATTGTTTT | 95ºC, 10 min.:(94ºC, 30 sec.: 50.5ºC, 30 sec.:  72ºC, 30 sec.) ×38 cycles: 72ºC, 7 min. | 10 | 257 bp |
|  |  | CR1 up1 CH3 R1 | CCCAAATACAACAACAAAACTTAAC |  |  |  |
| *CLU* | intron 1  S. Fig. 2 | clusterin intron1 CH3 F1 | TTTGTTGGAGGTATTTTAGGGTTTA | 95ºC, 10 min.:(94ºC, 30 sec.: 55ºC, 30 sec.:  72ºC, 30 sec.) ×40 cycles: 72ºC, 7 min. | 8 | 328 bp |
|  |  | clusterin intron1 CH3 R1 | ACCCCTCCCAAAAACTACTTATCTA |  |  |  |
| *CLU* | intron 1  S. Fig. 2 | CLU int1 CH3 F2 | GGGGTAGAGTTAGTGAGATTATAGTT | 94ºC, 10 min.:(95ºC, 30 sec.: 54ºC, 30 sec.:  72ºC, 30 sec.) ×48 cycles: 72ºC, 7 min. | 4 | 177 bp |
|  |  | CLU int1 CH3 biotin R1 | biotinylated-CCCATCTAAACACTCAATAACACAT |  |  |  |
|  |  | CLU int1 PyroSeq F1 | ATAGTTTTTTTGGTGAGTT | internal primer for pyrosequencing |  |  |
| *PICALM* | upstream  S. Fig. 3 | PICALM upst CH3 F1 | GTTTGAATGGGATTTTTTTATTTAT | 95ºC, 10 min.:(94ºC, 30 sec.: 55ºC, 30 sec.:  72ºC, 30 sec.) ×38 cycles: 72ºC, 7 min | 6 | 265 bp |
|  |  | PICALM upst CH3 R1 | ATCACCTAATTTTATTTATCTCTACTCC |  |  |  |
| *ABCA7* | upstream  S. Fig. 4 | ABCA7 up2 CH3 F1 | TTTTTTTTGTTATTTTTTGTAGAAGT | 95ºC, 10 min.:(94ºC, 30 sec.: 55ºC, 30 sec.:  72ºC, 30 sec.) ×36 cycles: 72ºC, 7 min. | 12 | 238 bp |
|  |  | ABCA7 up 2 CH3 R1 | ATCTTTAAAAAACCAAACTACCTCC |  |  |  |
| *BIN1* | upstream  S. Fig. 5 (region I) | BIN1 upst CH3 F2 | TTTTGTTGGGGGTAGGAGATAAT | 95ºC, 10 min.:(94ºC, 30 sec.: 55ºC, 30 sec.:  72ºC, 30 sec.) ×38 cycles: 72ºC, 7 min. | 9 | 209 bp |
|  |  | BIN1 upst CH3 R2 | ATAACAACAACCTAACATTACTAAATACTT |  |  |  |
|  | intron 1  S. Fig. 5 (region II) | BIN1 intron1 CH3 F1 | TTTGAAATTTGAGATTGTAGAATTTGT | 95ºC, 10 min.:(94ºC, 30 sec.: 55ºC, 30 sec.:  72ºC, 30 sec.) ×38 cycles: 72ºC, 7 min. | 9 | 159 bp |
|  |  | BIN1 intron1 CH3 R1 | ATTTATAAACAACCACCAAACCTAAC |  |  |  |
| *TREM2* | intron 1  S. Fig. 6 | Hs_TREM2_F | AAGGGGAATAAAGTTATAGAAATAGGG | 94ºC, 10 min.:(95ºC, 30 sec.: 58ºC, 30 sec.:  72ºC, 60 sec.) ×45 cycles: 72ºC, 10 min. | 4 | 179 bp |
|  |  | Hs_TREM2_R_bio | biotinylated-CCTCCAATTCTATTCTACACATCT |  |  |  |
|  |  | Hs_TREM2_S1 | ATAGGGAAGTTGGAAG | internal primer for pyrosequencing | | |
