# Supplementary material for: Lower DNA methylation levels in CpG island shores of CR1, CLU, and PICALM in the blood of Japanese Alzheimer’s disease patients
Source: PLoS One. 2020 Sep 29;15(9):e0239196. doi: 10.1371/journal.pone.0239196 (PMC7523949; doi:10.1371/journal.pone.0239196)
Supplement: S3 Table — (DOC) [file pone.0239196.s015.doc]

| dbSNP ID | Closest Ref Seq gene | Position (GRCh38.p12) | MAF (Ref./Alt.) | OR | 95% CI | *p*-value (two-sided) |
| --- | --- | --- | --- | --- | --- | --- |
| rs3818361 | *CR1* | chr1:207611623 | 0.435 (G/A) | 1.722 | 0.6547 to 4.530 | 0.3370 |
| rs11136000 | *CLU* | chr8:27607002 | 0.274 (C/T) | 0.9180 | 0.4991 to 1.688 | 0.8773 |
| rs3851179 | *PICALM* | chr11:86157598 | 0.347 (C/T) | 0.7421 | 0.3118 to 1.766 | 0.5169 |

AD; Alzheimer’s disease, NCGG; National Center for Gerontology and Geriatrics, MAF; minor allele frequency, OR; odds ratio, CI; confidence interval, *p*-value was calculated by Chi-square test.
