# Supplementary material for: Lower DNA methylation levels in CpG island shores of CR1, CLU, and PICALM in the blood of Japanese Alzheimer’s disease patients
Source: PLoS One. 2020 Sep 29;15(9):e0239196. doi: 10.1371/journal.pone.0239196 (PMC7523949; doi:10.1371/journal.pone.0239196)
Supplement: Attachment — Submitted filename: Response to Reviewers.docx [file pone.0239196.s018.docx]

**Answers to Reviewer #1**

1. As Reviewer #1 requested, we included the top six genes, *CLU*, *CR1*, *PICALM*, *BIN1*, *APOE*, and *ABCA7,* in the Abstract.
2. As Reviewer #1 requested, we cited appropriate references for the following sentence: “CpG islands are exempt from DNA methylation irrespective of gene expression, and the further away CpG dinucleotides are from a CpG island, the higher the chance of methylation.” in Introduction line 63. We consider it a well-known phenomenon as the references have been cited in many reports.
3. As Reviewer #1 requested, we described the reason for selecting Sanger sequencing, pyrosequencing, or by both methods in Materials and Methods line 104. As Sanger sequencing can quantify methylation levels of longer regions than pyrosequencing with in the same time, we selected it to identify the locations of CpG island shores in target genes. Sanger sequencing was again employed to find such CpG island shores where unidirectional changes in DNA methylation occur at consecutive CpGs. This was because DNA methylation changes are not at isolated CpGs but at consecutive CpGs in a gene accompanying biologically meaningful changes such as modification of chromatin and consequent alternation of gene activity. To confirm methylation changes identified by Sanger sequencing, we employed pyrosequencing because it is much higher throughput.
4. Contrary to Reviewer #1’s indication, “CyGnusPlotter” is not the program to predict possible locations of CpG island shores in a gene. It simply draws the positions of CpG dinucleotides along the gene of interest. We chose candidate CpG island shores in the plots by eye, judging from the density of the CpGs. They were not confirmed as CpG island shores until they were found to be slightly (10~20%) or moderately (~50%) methylated. This is described in the section of “Identification of CpG island shores” in the Materials and methods.

   Reviewer #1 wonders if the methylation changes in CpG island shores affect gene expression. We examined the possibility in a group (32) of our subjects and presented the data in the new Results section “Correlation between gene expression and methylation levels” (line 234) and in S8 Fig. No significant expression changes were observed with the sample set.
5. As Reviewer #1 requested, we explained the quality of pyrosequencing data in Methods line 120. Not all data passed the quality check and we reanalyzed failed data, which are shown as red scores by PyroMark Q48 Autoprep Software. We added the figure that shows the typical peaks of pyrosequencing (Pyrogram) in S7 Fig.
6. As the Reviewer #1 pointed out, we should have considered multiple comparisons for the statistical analyses of DNA methylation differences in Fig 2. We set *p*=0.00625 (0.05 divided by 8) as the threshold for a significant difference for the analyses and revised the figure accordingly.
7. As Reviewer #1 pointed out, we agree that it was inappropriate to select the top six known AD-related genes from the database, AlzGene, because it relies on the GWAS meta-analysis published in 2013. Instead, as Reviewer #1 suggested, we cited the latest GWAS meta-analysis data, in which the six genes we selected were included in the top seven AD-related genes. The Abstract, Results line 166, and Discussion line 370 were revised.
8. As Reviewer #1 requested, we described the absence of DNA methylation difference in *APOE*, *BIN1*, and *ABCA7* in Results line 204. We briefly discussed the negative results in Discussion line 374 and referred to the two papers that Reviewer #1 noted.
9. Reviewer #1 suggested that the number of *APOE* ε4 alleles should not be included as covariates if we want to demonstrate the impact of diagnostic values of methylation levels. Unfortunately, the diagnostic values of methylation differences alone in any of the three genes, *CLU*, *CR1*, and *PICALM*, fell far short of diagnostic purposes, as we described in Results line 340 in the revised manuscript. For blood-based AD diagnosis, we do not stick to DNA methylation differences alone and adopt anything that can be utilized for better diagnosis. In this sense, *APOE* genotyping provides ideal information because it comes from blood DNA and has high diagnostic value; therefore, we would like to utilize it in combination with methylation differences.

**An answer to Reviewer #2**

1. As Reviewer #1 requested, we added “Japanese” in the title of our manuscript.

**Answers to the eight comments, [A1] ~ [A8], in the Word file of the manuscript:**

[A1]: We added “Japanese” in the title as requested.

[A2]: We thank a reviewer for pointing out the mistake. We used peripheral blood leukocytes, not serum, and DNA was isolated. We corrected the mistake in Materials and methods line 89.

[A3]: We confirmed that the primers that were predicted to be useless in slico actually worked. The evidence is shown in S12 Fig.

[A4] & [A5]: we meant “slightly” and “moderately” for 10~20% and approximately 50%, respectively. To be clear, we also included the actual predicted percentages in parentheses in Results line 174 and in the Figure 1 legend line 195.

[A6]: We detected no significant difference at any single CpG site in the amplicons from the six genes, *ABCA7*, *APOE*, *BIN1*, *CLU1*, *CR1*, and *PICALM*, after Bonferroni correction, which was shown in S2 Table, and we described this in Discussion line 410. However, methylation differences with biological significance may be overlooked by Bonferroni correction for multiple testing. Using JASPAR 2020, we found that, among the CpG sites above, there are some with low *p*-values to which transcription factors can bind. We expect sufficient methods for multiple testing to be found ex post facto when changes in methylation affect transcription through such factors.

[A7]: As stated in Discussion line 380 in the revised manuscript, whether the methylation differences we found in AD blood are Japanese specific cannot be confirmed until they are examined in other ethnic populations. As the reviewer noted, a decrease in methylation at specific loci may be due to the decline in the DNA methyltransferase family, but to the best of our knowledge, no studies have revealed that it is a population-specific phenomenon or that any population-specific methylation differences occur in AD or any other diseases. We think it is one of the important issues to be addressed in our future studies.

[A8]: We appreciate this comment, which led us to a new finding. Stratification of *APOE* methylation by *APOE* genotype revealed a higher methylation level in *ε4* homozygotes, as shown in S9 Fig, suggesting the possibility that higher methylation at the *APOE* CpG island shore region and *APOE4* synergistically affect the onset of AD. We described the data in Results line 394.
